# Septicæmia after Amputations

**Published:** 1877-05

**Authors:** W. W. Jones

**Affiliations:** Toledo, O.


Septicæmia after Amputations.—*By W. W. Jones, M.D., Toledo, O.*—Septicæmia is, comparatively, a modern term, to represent blood poisoning. Most of the writers up to the present time have been content to use the term pyæmia, to express a condition which has been well known, under the supposition that it was produced by the absorption of pus.

The condition plays a most important part in disease, in obstetrics, in injuries, and in surgical operations.

Time and opportunity would fail me to dilate upon the general subject, and I shall only attempt to call your attention to its influence in amputations.

The practice of uniting the flaps after the amputation, for the purpose of obtaining immediate union, originated in England during the latter part of the last century, and has been termed the English method, in contradistinction to the plan followed by many of the French and other continental surgeons who believed that the wound should be allowed to granulate.

The great Frenchman, Baron Larrey, disapproved of the attempt to unite the wound after amputation by first intention, and very many of the French surgeons to this day follow his example; in opposition to which it has been said to be the pride of English surgeons, and an evidence of their superiority, that they had adopted the plan of bringing the edges of the wound together after amputation so that they may unite by the first intention.

These are great and radical differences of opinion, and as our methods in this country have been borrowed from England, it is especially pertinent to investigate the subject, in order that we may come to an understanding of the truth. It cannot be questioned that Baron Larrey had a larger clinical experience than any other man who had lived before or even since his time; and it is also true that the utmost confidence was placed in his skill and integrity by the people of France, and the profession of the civilized world. He placed great stress upon the necessity of free drainage until healthy granulations had commenced; for, he contended, *they did better*, and we have no reason to suppose that he obtained this opinion except from his clinical experience, and the English practice was as well known to him as to us. The great German surgeon Chelius, in his work on surgery, says: "Many practitioners have exaggerated the evils accompanying the cure by suppuration and granulation, for the wound after amputation of large limbs never takes place by complete agglutination in the strict sense of the word."

Gross says that "the most common constitutional occurrences after amputations, especially of the larger limbs, are excessive prostration, traumatic fever, pyæmia, congestion of the lungs, retention of urine, and as a secondary consequence, hectic irritation."

In treating of the danger after operations, this justly celebrated author says: "More or less fever must almost necessasarily follow every severe operation, offering thus an additional source of suffering and danger to the patient, and of anxiety to the surgeon.

"To this disease the term traumatic is usually applied; the older writers called it bed or wound fever, and Dr. Simpson has given a most excellent account of it under the name of surgical fever.

"It generally begins within the first six or eight hours after the operation, and is characterized by a flushed appearance of the face, a frequent, quick, and irritable state of the pulse, dryness of the skin, restlessness and thirst, which is often excessive, especially after profuse losses of blood. In some cases there is extreme jactitation, with nausea, if not actual vomiting, and a tendency to delirium, or a confused and bewildered condition of the intellect.

The breathing is generally somewhat hurried, and slight mucous rales are often present. The appetite is impaired or entirely wanting, the bowels are often constipated and the urine is scanty, high-colored, and often offensive, being surcharged with saline matters, and occasionally even slightly albuminous. Now and then the disease is ushered in by distinct rigors. After the fever has continued for some time, a tendency to remit appears; the heat, thirst, and restlessness diminish, the pulse descends, the gastric distress vanishes, and the skin becomes covered with a gentle moisture, which often increases to profuse perspiration.

"The duration of traumatic fever varies from a few hours to a number of days. In the milder cases it is generally very evanescent, while in the more severe it is often quite protracted, and is then usually attended with regular vesperal, if not also matinal, exacerbations.

"Frequently the disease disappears entirely for a few days, and then recurs, with more or less violence, the attack being provoked either by some dietetic or other indiscretion on the part of the patient, or by some change of the system, as the arrest of an important secretion, or the commencement of blood-poisoning.

"The causes of surgical fever are sufficiently obvious. Every operation of the slightest severity acts as a disturbing agent, depressing the vital powers, and deranging as a direct and inevitable consequence, all the secretions. We have familiar illustrations of these occurrences in daily practice, in the lancing of boils, the extraction of teeth, and in the operation of bleeding. The least shock, however induced, is sure to be followed by more or less general disorder, and it is hardly necessary to state that the amount of this disorder is always materially augmented when, in addition to the nervous lesion, there has been considerable loss of blood. Nothing so rapidly or so powerfully irritates and frets the vascular and nervous systems as these two circumstances. The heart evinces its suffering first, by the feebleness and irregularity, and, after the occurrence of reaction, by the frequency and rapidity of its contractions. The nausea and vomiting, the excessive prostration, the yawning and confusion of intellect, not to mention other symptoms, are unmistakable signs of the shock, or loss of power, sustained by the great nervous centers. The use of anæsthetics, doubtless, often contributes to the production of traumatic fever, by the disturbance which it occasions in the general system.

"Now, if we take all these circumstances into view, and the fact that thousands of patients are subjected to operations without due preparation of the system, or, indeed any preparation at all, as in cases of primary amputations, trephining, and the ligation of the larger arteries for the arrest of hemorrhage in gunshot and other wounds, *it is certainly not necessary to invoke the agency of blood-poisoning, as is of late frequently done, to account for the occurrence of traumatic fever.* These causes are all sufficient to create the most violent disturbance, or, in other words, to set the whole system in a perfect state of ferment, overturning all its functions, and thus occasioning an amount of reaction capable of destroying life in a few hours, or at all events, in a few days. While this perturbation is progressing, other and still more serious consequences may ensue, as erysipelas, pyæmia, and effusions into the splanchnic cavities, the result of disordered secretion and of the retention of hurtful matter, eventuating in a diseased state of the blood, and in a predisposition to local inflammation in parts more or less remote from the seat of the original injury. The constitutional derangement will, of course, be materially increased if, in addition to these disturbing agencies, there is an absorption or ingress of foul pus into the system, constituting toxæmia, or blood-poisoning."

This description of surgical or traumatic fever after amputation given by Dr. Gross, will not vary greatly from such a case of septicæmia after ovariotomy, either in its symptoms or results, as might be detailed by a number of surgeons whom I could mention, and probably by some who have just listened to it.

When we take into consideration the fact that the sero-sanguineous exudation, which drains from an amputation wound, is subjected to a heat of not less than 95° Fahrenheit, and in six hours has a strong, and in twenty-four hours a decidedly bad odor, and also the fact now well known, that absorption from this surfrce is active and efficient, it is no wonder that the agency of blood-poisoning should be invoked to account for the occurrence of traumatic fever.

It will not do to charge it to the loss of blood, as intimated in the quotations from this author, because the well known phenomenon accompanying this symptom in parturient subjects is totally unlike that witnessed by the surgeon in cases referred to, and I will not detain you by going into a detail of the differential symptoms which go to make up the two distinct classes of phenomena.

Wagner, in his general pathology, says that "all the phenomena of traumatic fever in man are best explained by the hypothesis that it is dependent upon inflammations set up, not by the injury itself, but by septic processes taking their start in the wound.

"It is most probable that the fever is caused only by spores which enter the blood through the wound, and that sepsis is the result of vibrionic putrefaction. To what degree these inflammations of the wound and the fever dependent thereon are developed, or whether they develop at all, depends upon the nature of the wound, the mode of dressing, the state of the atmosphere in which the patient is, the mechanical factors which favor the entrance of putrid substances into the tissues and the blood, upon the quality of the putrid substances, intensity of their inflammation—producing (phlogogenic) and heat—producing (pyrogenic) activity, etc. Consequently, if immediately after a wound or operation the temperature suddenly rises, it is possible that an infection, direct or indirect, of the wound has occurred, or that inoculation with a pyrogenic substance has taken place. Among other means, the quick removal of the infecting inflammatory focus is necessary in order to bring the fever to an end.

"The following account of the simple fever which accompanies wounds—'traumatic fever'—is sufficient for clinical purposes:

"Traumatic fever lasts from one to seven days and more, without any tendency to cease on any particular day; it is continuous with the secondary fever (after the seventh day) in case of severe injury, and even in cases of accidental inflammation and suppuration. The highest temperature is attained in traumatic fever on the first or second day, seldom from the third to the fifth day, very rarely later; still the temperature may rise higher in the secondary fever.

"In septicæmia the temperature is, as a rule, very high in the beginning, and later becomes very low; though there are cases in which it is low at the commencement and never rises very high; it sometimes hardly exceeds the normal.

"Death may occur at any point of these oscillations of temperature, or it may follow a rising high temperature, or one falling far below the normal. In its course chills are exceedingly rare."

The experiments of Billroth, Weber, and Bergmann, by injection of putrid substances under the skin and into the veins of animals, are interesting as confirming the premises laid down by Wagner.

Billroth does not hesitate to declare in his most recent work that "traumatic and inflammatory fevers arise through the absorption of pyrogenic substances, produced upon, or in the wound, or from such as are deposited upon it. The intermissions of traumatic fever chiefly depend upon the intermittent taking of the infecting body into the blood; and that chills were more dependent upon the absorption of *purulent* than upon that of *ichorous matter.*"

I think the experience of every surgeon will bear me out in the statement that a large majority of the cases that die during the first week after amputation, are attended with surgical fever, and that it is during this period that absorption and septic activity must prevail. After suppuration has commenced the phenomena are to a considerable extent changed, and complications, believed to be due to emboli and hectic, make their appearance, and produce death by a combination of causes which may be traced to the joint action af septic and pyæmic causes.

It is only within a very few years that attention has been given by the profession to the prevention of septic influences. In this country it has been greatly promoted in its surgical aspects by the experience of the late war of the rebellion. Mr. Lister, of Edinburgh, has achieved an enviable success by means of the carbolic spray and strict attention to the details of cleanliness.

Professor A. B. Crosby, of Bellevue Hospital, in a recent paper read before the New Hampshire State Medical Society (1876), speaks of cleanliness as "one of the lost arts to be restored in surgery."

The paper abounds in facts and clinical data upon the subject of septic poisoning, and the means to be made use of to prevent or overcome it, so that one cannot help being convinced that the utmost vigilance and care, in respect to cleanliness, will well repay the surgeon for his efforts. It will no longer do for the surgeon to use the sponges, bandages, bedding, and linen which may have been previously about another patient, no matter how well it may have been washed. The room itself that has been used by a patient with septic disease becomes a receptacle of foci, which may propagate disease in the unfortunate victim who next occupies it.

Recently the practice of leaving wounds open after amputation has been adopted in some of the surgical wards of Bellevue Hospital, by Dr. James R. Wood, thus affording ample drainage until a healthy granulating surface has been obtained. He uses the pillow of oakum, irrigations of carbolic acid water, and has the open wounds smeared with balsam peru, and requires that no hand or instrument shall come in contact with a wound without first being washed in carbolized water or a solution of chlorinated antiseptic.

Dr. Dennis, house surgeon of Bellevue Hospital, details, in a recent number of the *New York Medical Journal*, ten cases of amputation of the thigh, leg, arm, and knee, by Dr. Wood. The cases were generally those of compound comminuted fracture, in patients of intemperate habits, and the results were rapid recovery without a death; very mild suppurative fever, in which the temperature never exceeded 103°, but was more frequently much lower; in no case was there erysipelas of the stump, or abscess, or sloughing, and the resulting stump was very satisfactory in form and for the purposes of an artificial limb.

My time and space will not permit me to quote from the papers of Dr. Crosby and Dr. Dennis, which must be read to be appreciated.

Our surgeons in the late war of the rebellion reported unexpectedly favorable results when they were unable to close the wound after amputation, either from want of materials with which to do it, or other causes beyond their control; and they can only account for such good results upon the hypothesis that there was less opportunity for blood-poisoning by the absorption of septic material from an open wound, than would have been the case had attempts been made to close them up.

The treatment of amputation wounds without closing, so far as trials have been made in this country, have proved eminently satisfactory, and would seem to demonstrate conclusively that our previous methods (borrowed from the English) of closing the flaps so that it may heal by first intention, instead of being an advantage, is really prejudicial; and that we must admit that the older continental surgeons were right in refusing to be convinced against their experience, or if I may be permitted to use a word heretofore unknown in medicine, *bulldozed* into admitting that the English practice was the best.

The exact value of antiseptics in the treatment of open wounds has not yet been very fully tested; nor are we yet able to fully explain the causes of septicæmia, or whether it is connected with bacteria or other minute organisms, or is analogous to the poisonous fluids of infective diseases. Still enough is known to establish beyond a doubt that they are essential to the prevention of putrefaction, which if permitted to go on, may not only poison the vital fluid, but as all surgeons know, powerfully depress the nervous system.

From what has been said, it follows that the patient, after amputation, must not be poisoned by the absorption of his own excretions or exudations, even if it overturn the long cherished pride of English surgeons in their adopted plan of bringing the edges of the wound together after amputation, so that they may unite by the first intention.

The use of sutures and adhesive plasters must be discarded if we would prevent absorption of those elements which nature casts out of a wound made by amputation. It is too late now to say that public opinion may not sustain the surgeon in what may very properly be called *an air dressing;* for public opinion may be in the condition that Martin King once found himself. A good many years ago Martin had a compound fracture of the leg near the ankle, and I was compelled to saw off more than an inch of the end of the tibia in order to reduce it. It was hot weather, in July, and the flies were thick. One morning early a messenger routed me out of bed to go and see him, as he thought he would soon die. On arriving at his bedside, King gravely told me that the maggots had been going up inside of him all night, and had now got into his brain; "he could feel them there, be jabers, no mistake." Martin King has since become convinced that he *was mistaken.*—*Toledo Med. and Surg. Jour.*

